# Impact of Mild Hypohydration on 100 m Front Crawl Performance and Starting Block Peak Force Production in Competitive University-Level Swimmers

**DOI:** 10.3390/sports8100133

**Published:** 2020-10-14

**Authors:** Mohamed El Fethi Abed, Thomas A. Deshayes, Pascale Claveau, David Jeker, François Thénault, Eric D.B. Goulet

**Affiliations:** 1 Faculty of Physical Activity Sciences, University of Sherbrooke, Sherbrooke, QC J1K 2R1, Canada; Mohamed.El.Fethi.Abed@USherbrooke.ca (M.E.F.A.); Thomas.Deshayes@USherbrooke.ca (T.A.D.); Pascale.Claveau2@USherbrooke.ca (P.C.); David.Jeker@USherbrooke.ca (D.J.); Francois.Thenault@USherbrooke.ca (F.T.); 2 Research Centre on Aging, University of Sherbrooke, Sherbrooke, QC J1H 4C4, Canada

**Keywords:** anaerobic performance, euhydration, hydration, hypohydration, swimming

## Abstract

Unstructured, ad libitum drinking may predispose some athletes to start exercise already slightly hypohydrated (decreased body water). The impact of pre-exercise mild hypohydration on subsequent swimming performance is still unknown. Hence, the goal of this study was to examine its effect on peak force production on the starting block and 100 m front crawl swimming performance in competitive university-level swimmers. At least one hour after having been passively exposed to heat where a body mass loss of 1.5% was induced or euhydration (normal body water) maintained, nine participants (age: 22 ± 2 years) underwent an assessment of their peak force production on the starting block and 100 m front crawl performance. One hour following hypohydration, rectal temperature had returned to baseline in each condition. Urine osmolality and specific gravity were higher (*p* < 0.05) with hypohydration than euhydration (995 ± 65 vs. 428 ± 345 mOsmol/kg; 1.027 ± 0.003 vs. 1.016 ± 0.007 g/mL) prior to exercise testing, as was perceived thirst. Swimming performance (*p* = 0.86) and peak force production (*p* = 0.72) on the starting block did not differ between the hypohydration and euhydrated condition (63.00 ± 4.26 vs. 63.09 ± 4.52 s; 1322 ± 236 vs. 1315 ± 230 N). The current results indicate that mild hypohydration, which may occur with ad libitum drinking, does not impede peak force production on the starting block and 100 m front crawl performance in university-level competitive swimmers. Planned drinking is not required prior to such an event.

## 1. Introduction

Swimming has been on the program of the Summer Olympic Games since the first edition of the Games in 1896 [1] and is an extremely popular sport throughout the world. Among all events, the 100 m front crawl is often considered to represent the blue-ribbon race in competitive swimming. At the present moment, the 100 m front crawl long-course world record is 46.91 s, with the second and third place finishers trailing by only 0.21 and 0.34 s, respectively. The fourth place finisher missed the first and third place of the podium by only 0.36 and 0.02 s, respectively [2]. This event requires a combination of muscle power and strength to produce a high take-off horizontal velocity at the starting block, which plays a critical role in 100 m swimming performance [3,4], and muscle anaerobic capacity and endurance to maintain a high swimming velocity throughout the distance [5,6,7].

It is generally not the habit of athletes to structure their intake of fluid before exercise, and ad libitum drinking may occasionally cause hypohydration (decreased body water) prior to exercising [8,9]. Competitive adolescent [10,11], as well as adult [12,13] swimmers have been demonstrated to report to the training pool for a training session already hypohydrated, based on urinary markers of hydration, i.e., with a urine specific gravity (USG) ≥ 1.02 g/mL or urine osmolality (Uosm) ≥ 700 mOsmol/kg [14]. Although unknown, these observations suggest that some competitive swimmers may also engage in a competition hypohydration. Moreover, it is not a rare occurrence that swimmers compete in multiple events during a swimming contest. The excitement of the day combined with the multiple cool-downs, warm-ups and changing periods may provide insufficient time to reverse hypohydration, putting the athlete under a situation where he/she may participate in more than one swimming event hypohydrated. Therefore, the impact of pre-exercise hypohydration in swimmers is not to be mitigated and requires attention.

The impact of hypohydration on high-intensity muscle performance has been mostly examined during land-based exercise. [15,16]. Two studies have looked into the effect of drinking on repeated 50 or 100 m swimming performances during a regular training session. Whereas Taimura et al. [17] showed that drinking water during training improves swimming performance, compared with no fluid intake, Briars et al. [18] did not. No study has yet examined the impact of pre-exercise hypohydration on a single distance event recognized by the International Swimming Federation (FINA).

In a meta-analysis, Savoie et al. [16] reported that hypohydration of on average 3% body mass decreases anaerobic power and strength by 6%, as well as muscle endurance by 8%. Jones et al. [19] demonstrated that hypohydration of 3% body mass reduces upper-body 30 s Wingate performance by 7%, compared with euhydration (normal body water). A potential limitation of those studies is that most produced hypohydration levels >2% body mass, which are unlikely to be representative of the true state of hypohydration in which an athlete may enter a training session or competition. Indeed, it is reasonable to assume that athletes would not let themselves reach a relatively strong and persistent thirst sensation prior to training or a competition [20,21], and this threshold usually sets in at a hypohydration level ≥2% body mass [22]. A state of hypohydration of 1.5% body mass, which is higher than the normal daily variation in body water (i.e., ≤1% [23]), yet lower than the thirst threshold, may represent a reasonable and realistic hypohydration level encountered by athletes during free-living conditions while drinking ad libitum.

Given the substantial impact that hypohydration may have on muscle performance vs. the tight margins of time for podium exclusion, understanding the degree to which hypohydration could impact a swimmer’s performance is essential for the athletes, coaches and sports dietitian looking for optimal race preparation and performance. Therefore, the aim of this study was to examine the effect of mild hypohydration (equivalent to 1.5% body mass loss) on 100 m front crawl performance and starting block peak force production in university-level competitive swimmers. We hypothesized that 100 m front crawl performance and starting block peak force production would be impaired by mild hypohydration.

## 2. Materials and Methods

### 2.1. Participants

Healthy swimmers (7 men and 2 women) from the University of Sherbrooke swimming team participated in this study, which was approved by the University of Sherbrooke Institutional Review Board. All experimental procedures were explained, and the participants provided written informed consent before the preliminary visit. The participants’ physical, physiological, anthropometric and training characteristics are presented in Table 1.

### 2.2. Experimental Design

After a preliminary visit, participants took part in: (1) three familiarization trials (every three days); and (2) two experiments (spaced by seven to ten days), which consisted of blocks of passive heat exposure to induce a dehydration of 1.5% body mass through sweat loss, followed by a 1 h passive recovery period and then a testing session to evaluate, first, the force produced by the lower limbs on the starting block and, second, the 100 m front crawl performance. Both the familiarization trials and experiments always started at the same time of day in the afternoon (1:00 pm). Hydration conditions were evaluated using a randomized, crossover and counterbalanced study design. Women underwent the experiments during the follicular phase of their menstrual cycle, i.e., the period ranging from the first day of menstruation + the following 13 days.

### 2.3. Preliminary Visit

During the preliminary visit, participants’ physiological, physical, training and anthropometric characteristics were assessed. Training characteristics were determined with a questionnaire. Post-void body mass was measured to the nearest 20 g using an electronic balance (BX-300+, Atron Systems, West Caldwell, NJ, USA) and height using a wall stadiometer. Both fat mass and lean mass (LM) were assessed with dual-energy X-ray absorptiometry (Lunar Prodigy, GE Healthcare, Madison, WI, USA). After a period of 3 min of seated rest, resting heart rate and blood pressure were measured using an automatic sphygmomanometer (Welch Allyn, Skaneateles, NY, USA).

### 2.4. Familiarization Trials

These trials were performed to familiarize the participants with all the experimental procedures and to reduce any learning effect that usually becomes negligible after two familiarization trials [24]. A third familiarization trial was performed to calculate the coefficient of variation (CV) of the different performance tests between the second and third familiarization trial. For each of the familiarization trial, participants first underwent a standardized 30 min warm-up period, then three jumps on the force plate each interspaced by a 2 min recovery period and, finally, after a last a 2 min recovery period, the 100 m front crawl.

### 2.5. Pre-Experimental Procedures

Participants kept and filled out a fluid and diet log over the 24 h prior to the first familiarization trial and then replicated it over the last 24 h prior to the remaining familiarization trials and experiments. Participants consumed 250 mL of water 60 min prior to bedtime, which was standardized prior to all familiarization trials and experiments. The same amount of water was also consumed 60 min prior to all familiarization trials and experiments. Participants were allowed to consume caffeine-containing products to prevent withdrawal-associated symptoms; however, the amount consumed within participants was always similar prior to the familiarization trials and testing periods. Consumption of dietary supplements and strength training were forbidden for 48 h prior to all familiarization trials and experiments. Throughout the study period, participants were asked to keep their normal training routine, except during the last 8 h prior to the familiarization trials and experiments, where they were requested to refrain from training.

### 2.6. Experimental Procedures

#### 2.6.1. Arrival at the Laboratory

At arrival, participants provided a urine sample (for USG and Uosm analyses), and their post-void body mass was taken with only their competitive swimsuit on. The mass of the swimsuit was then subtracted to obtain a baseline body mass. This body mass was taken as the nude, baseline, euhydrated body mass from which a 1.5% hypohydration level was calculated. They then inserted a telemetric probe (CorTemp, HQ Inc, Palmetto, FL, USA) just passed the anal sphincter [25]. Before entering the environmental chamber, maintained at 45 °C and 20% relative humidity (RH), and while wearing only a swimsuit, rectal temperature was measured, and participants were asked to provide their perceptions of thirst [26] and heat stress [27].

#### 2.6.2. Heat Exposure and Hypohydration

Body mass loss was induced by passive sweating, while alternating between blocks of 25 min of seated heat exposure and 2 min recovery blocks outside the environmental chamber (~20–21 °C and 30% RH), until participants had accumulated a loss of body mass of 1.5%. A passive, not active dehydration technique was chosen to not induce any muscle fatigue prior to exercise, thereby maximizing the validity of the performance outcomes. Moreover, in comparison to active dehydration, passive dehydration reduces the release of metabolic water into the body water pools, thereby enabling obtaining a tighter relationship between body mass loss and body water loss. Induction of hypohydration the evening before the experiments was ruled out to not disrupt athletes’ sleeping quality because of thirst. Water restriction only the day before the experiments was deemed not to be an effective strategy due to the difficulty in effectively controlling hypohydration level [28].

While inside the environmental chamber, measurements of rectal temperature, perceived thirst and heat stress were taken at min 24 of each block. During the recovery blocks, participants voided their bladder and dried themselves with a dry-clean towel, and measurements of body mass were taken with participants wearing their swimsuit only. Body mass was always corrected for the mass of the swimsuit. However, a pilot study showed that swimsuits retained an insignificant amount of moisture during the heat exposure periods. Shortly (2 min) after re-entering the environmental chamber, participants received an amount of water (provided at 35 °C) equivalent to that lost through sweat (euhydration condition only) and urine (euhydration and hypohydration conditions) during the preceding heat exposure block and recovery period. If no urine was produced, participants in the hypohydration condition were allowed to rinse their mouth (to reduce dry mouth and thirst sensation) with 25 mL of water.

#### 2.6.3. Recovery Period

Following heat exposure, participants remained seated for 60 min in a room held at ~20–21 °C with 30% RH. This passive recovery period aimed to decrease core temperature to values comparable to their individual baseline level. At the end of the recovery period, participants voided their bladder, collected a midstream urine sample, were weighed and had their rectal temperature measured. They were then provided water in an amount equivalent to their accumulated body mass loss during the recovery period, to restore euhydration or the 1.5% body mass loss.

Following the recovery period, participants moved to the swimming pool, which was ~200 m away from the laboratory, and then completed a standardized warm-up consisting of 15 min of dry land stretching and dynamic exercises followed by 15 min of exercises in the water where participants made laps while controlling their own speed (total volume of 800 m performed at low- to high-intensity). Following the warm-up, participants voided their bladder, collected a midstream urine sample, were weighed, had their rectal temperature measured, removed the rectal telemetric probe and then, after 2 min of recovery, underwent measurements of their force produced on the starting block followed, after 2 min of additional rest, by the 100 m front crawl.

### 2.7. Measurements

Urine specific gravity was determined using a digital refractometer (PAL-10S, Atago, Bellevue, WA, USA) and Uosm with the freezing point depression technique (Micro Osmometer, Osmette, Precision Systems Inc., Natick, MA, USA). The force produced on the starting block was measured with an AMTI portable force plate [29] (OR6-6-1000, Watertown, MA, USA) calibrated before each experiment, which was then fitted and adapted to the starting block to keep the standard height from the water (0.7 m) and inclination (10º). The forces recorded by the force plate were first transformed to correct for the force plate inclination. Then, the peak resultant force (PFres) was calculated using the vertical (Fver) and horizontal (Fhor) components as indicated in West et al. [4], using the following equation:

(1) $$\mathrm{PFres}=\sqrt{\left({\mathrm{Fhor}}^{2}+{\mathrm{Fver}}^{2}\right)},$$

Participants used a rear-weighted kick start position. The 100 m front crawl was carried out in the first swimming lane of a 50 m swimming pool. Water temperature was maintained at 28 °C. The performance times were obtained in accordance with FINA regulations by averaging the times measured by three timekeepers, which were placed immediately beside the starting block to have an unobstructed view of the wall. The start signal was given by a qualified official, in accordance with FINA regulations, as follows: (1) on the whistle, swimmers climbed on the starting block; (2) the starter sent the command “Take your marks”; (3) the swimmers positioned themselves and kept a stationary position; (4) the starter called the starting signal “Go”.

## 3. Statistical Analysis

Normally distributed data (assessed with a Shapiro–Wilk test) were analyzed using either paired *t*-tests, one way repeated measures analysis of variance (ANOVA) or two way (condition × time) repeated measures ANOVA. In cases where sphericity was violated, Greenhouse–Geisser corrections were applied. A Wilcoxon signed ranks test, using the asymptotic test to calculate significance, were used for abnormally distributed data. Because not all participants could produce urine at all collection points, urine-related variables were analyzed using a linear mixed-effects model. When statistically significant time or interaction effects were detected, multiple pairwise comparisons were performed and corrected with the false discovery rate procedure. An intraclass correlation coefficient was measured to assess inter-rater reliability for the 100 m front crawl performance time. With nine participants, the probability to detect a statistically significant condition effect for the 100 m front crawl performance was 80%, based on a typical error of measurement of 0.3 s (corresponding to that measured between the second and third familiarization trial) and a minimal difference between conditions of 0.45 s (corresponding to 1.5 times the typical error of measurement). Statistical analyses were performed using the IBM SPSS Statistics software (Version 21, New York, Armonk, NY, USA). The threshold for statistical significance was set at 95% (α ≤ 0.05). Results are presented as means ± the standard deviation (SD).

## 4. Results

### 4.1. Participants’ Hydration Status at Arrival to the Laboratory

Participants were adequately and similarly hydrated before each experiment, as supported by the lack of difference in USG (1.016 ± 0.004 vs. 1.016 ± 0.004 g/mL, *p* = 0.63), Uosm (507 ± 180 vs. 503 ± 192 mOsmol/kg, *p* = 0.91), urine production (219 ± 18 vs. 218 ± 27 mL, *p* = 0.91) and body mass (71.2 ± 8.2 vs. 71.3 ± 8.9 kg, *p* = 0.65) between the euhydrated and hypohydrated condition.

### 4.2. Heat Exposure Duration and Hydration Status

**Figure 1:**
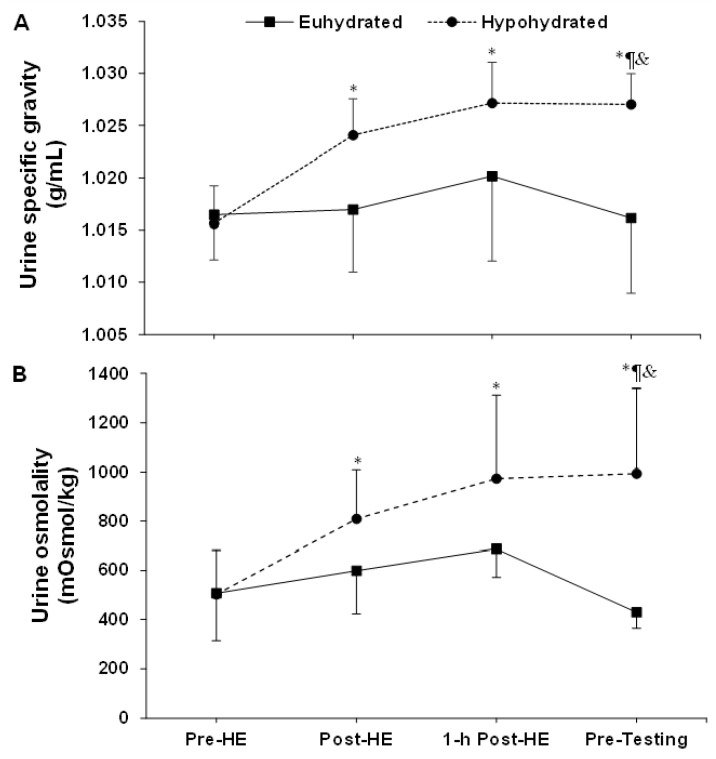
Changes in urine specific gravity (**A**) and osmolality (**B**) during the experiments. *: time effect; ¶: condition effect; &: interaction effect. Pre-HE: pre-heat exposure; Post-HE: post-heat exposure. Results are means ± SD.

The time to achieve the targeted loss of body mass, which takes into account the recovery periods, was 99 ± 13 min and 102 ± 22 min (*p* = 0.68) for the euhydrated and hypohydrated condition, respectively. Figure 1 shows the changes in USG (A) and Uosm (B) throughout the experiments, and as expected, a time, condition and interaction effect (all *p* < 0.01) was observed for both variables. Prior to starting the testing periods, USG was 1.016 ± 0.007 g/mL and Uosm 428 ± 345 mOsmol/kg with the euhydrated condition, compared to 1.027 ± 0.003 g/mL (*p* < 0.01) and 995 ± 65 mOsmol/kg (*p* < 0.01) with the hypohydrated condition, respectively.

### 4.3. Rectal Temperature

**Figure 2:**
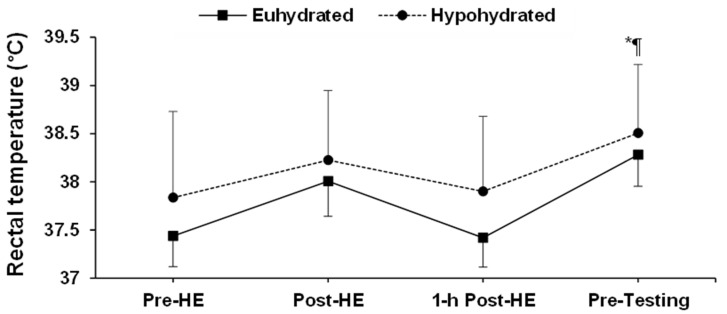
Changes in rectal temperature during the experiments. *: time effect; ¶: condition effect. Pre-HE: pre-heat exposure; Post-HE: post-heat exposure. Results are means ± SD.

The changes in rectal temperature during the experiments are illustrated in Figure 2. There was a condition (*p* = 0.02) and time (*p* < 0.01), but no interaction (*p* = 0.87) effect between hydration conditions. Following the 1 h recovery period, rectal temperature had returned to baseline for both hydration conditions (*p* = 0.91).

### 4.4. Perceived Thirst and Heat Stress

Perceived thirst and heat stress data are reported in Table 2. A time (*p* < 0.01), condition (*p* < 0.01) and interaction (*p* = 0.04) effect was observed in the change in perceived thirst between hydration conditions. Following heat exposure (*p* = 0.04) and prior to testing (*p* = 0.03), perceived thirst was higher while hypohydrated than euhydrated. On the other hand, a time (*p* < 0.01), but no condition or interaction effect was observed between hydration conditions for perceived heat stress.

### 4.5. 100 m Front Crawl

**Figure 3:**
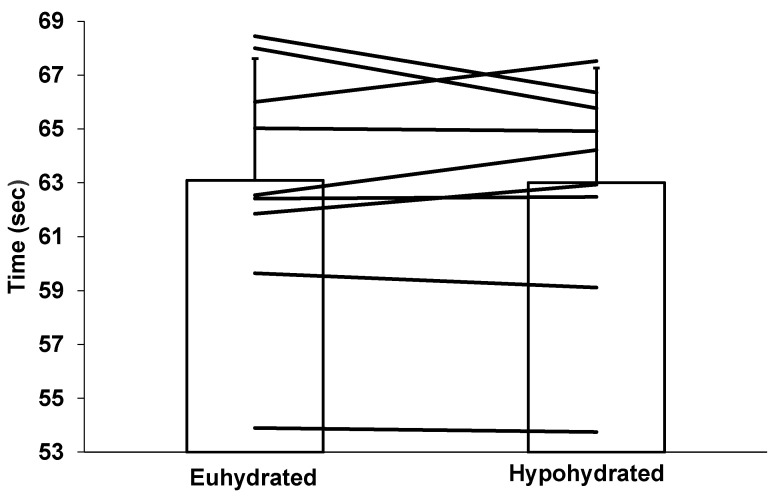
Performance during the 100 m front crawl for the euhydrated and hypohydrated condition. Horizontal lines represent individual changes in performance. Vertical columns represent mean performances with SD.

There was no order effect (*p* = 0.52) in performance. The inter-rater reliability for the 100 m front crawl time measurement was perfect at *r* = 1.00. There was no difference in 100 m front crawl performance between the euhydrated (63.09 ± 4.52 s) and hypohydrated (63.00 ± 4.26 s) condition (*p* = 0.86), as demonstrated in Figure 3. The day-to-day CV for the 100 m front crawl performance was 0.38%, whereas the percent change in performance between conditions was 0.13 ± 2.15%.

### 4.6. Peak Force Produced on the Starting Block

**Figure 4:**
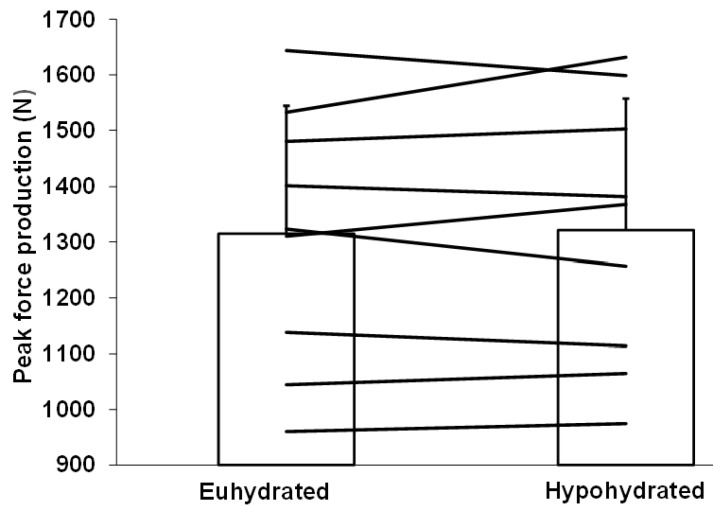
Absolute peak force production on the starting block for the euhydrated and hypohydrated condition. Horizontal lines represent individual changes in performance. Vertical columns represent mean performances with SD.

No order effect was observed for this variable (*p* = 0.61). Figure 4 demonstrates the impact of hydration conditions on the absolute peak force produced on the starting block. No difference was observed between conditions whether force production was corrected (hypohydration: 62.9 ± 9.4; euhydration: 62.6 ± 8.8 N/kg LM) or not (hypohydration: 1322 ± 236; euhydration: 1315 ± 230 N) (both *p* = 0.72) for lower limb LM. Correcting for pre-testing body mass level did not change the outcome either (*p* = 0.24). The day-to-day CV for absolute peak force produced on the starting block was 3.8%, whereas a difference of only 0.38 ± 3.6% was observed between hydration conditions for peak force production.

## 5. Discussion

This study examined the impact of mild hypohydration (1.5% body mass) on 100 m front crawl performance and peak muscle force production on the starting block in university-level competitive swimmers, and to the best of our knowledge, this is the first study to examine the effect of hypohydration on performance within this context. From a statistical perspective, none of the performance-related outcomes were shown to be negatively impacted by hypohydration. Moreover, as the changes in 100 m front crawl performance and peak muscle force production on the starting block between conditions were within the expected normal daily variation, it is unlikely from a practical standpoint that hypohydration would hinder these performance parameters.

Mild hypohydration can develop insidiously in an individual drinking ad libitum due to a slight mismatch between fluid intake and fluid losses through sweat, urine and respiration. In turn, blood hypertonicity will enhance ADH production, resulting in an increased urine solute concentration. Urine osmolality and specific gravity measured immediately prior to the testing period indicated that swimmers were indeed hypohydrated. The magnitude of urinary solute concentration compared favorably well with that observed by Adams et al. [10] and Arnaoutis et al. [11] in adolescent swimmers reporting to the pool for a training session. Hence, our dehydration protocol was successful in reproducing the mild hypohydration level that could develop under daily free-living conditions in swimmers.

The quality of a 100 m front crawl swim completed on a long-course depends on several components, including the start on the block, the flip turn, which comprises a push from the wall, underwater swimming, which is limited to 15 m/lap, and the swimming portion of the swim per se (clean swimming) [30]. Any variation in the quality of one or several of those components could result in a worthwhile change in performance, if uncompensated by other components. Hence, to be able to detect the potential small change in performance associated with hypohydration, we needed first to determine whether our participants were reliable in their ability to reproduce their 100 m front crawl performance. Our results indicate that they were as a group, as the performance CV from the second to the third familiarization phase was 0.38%, translating to an average change in performance of only 0.01 s.

The force applied by the lower limbs on the starting block plays a critical role in sprint-type swim races [31,32]. Indeed, it provides powerful momentum for the initial propulsion in the water. Moreover, air traveling generates less resistance compared with water [3]. Our results show that the peak force production on the starting block did not differ among hydration conditions. Additionally, the flip turn is associated with a complete change in direction, and the push off from the wall is pivotal in generating the final velocity to complete the last segment of the race [33]. It has been reported that the velocity created following the flip turn can be a determinant factor for medal standing [34]. Although not directly measured, it is reasonable to believe that the force applied to the wall by swimmers during the flip turn was also unimpacted by hypohydration. It follows, then, that if the block and push off phases from the wall were unimpacted by hypohydration, that the swimming portions per se, i.e., the clean swimming and dolphin style swimming underneath water, were also not.

We can only speculate as to why we were not able to detect an impact of mild hypohydration on 100 m front crawl performance. Men and women were combined, and some may believe that this could have mixed findings. However, both genders responded similarly to the change in hydration levels. Moreover, the % fat mass (men range: 6.9 - 27.5%; women range: 20.2–27.7%), % LM (men range: 71.4–92.8%; women range: 71.3–78.9%) and body mass index (men range: 20.2–27.6 kg/m^2^; women range: 21.0–25.4 kg/m^2^) were similar between some of the men and women. On the other hand, hypohydration level may have been potentially sufficient to impact performance, but the >1 h recovery period between the end of hypohydration and onset of testing may have allowed sufficient time for optimal re-equilibration of fluid between compartments, thereby minimizing any possible impact through cardiovascular, metabolic, buffering or neuromuscular mechanisms [15].

In a meta-analysis, Savoie et al. [16] demonstrated that hypohydration of on average 3% body mass impairs lower body muscle strength and endurance. Lower limb strength has been shown to be a key determinant for peak horizontal and vertical force production on the starting block [4]. Indeed, in male international sprint swimmers in whom peak resultant force produced on the starting block was on average 1545 N, in comparison to 1319 N in the current study, peak horizontal and vertical force production were highly correlated with 1 RM squat strength [4]. Moreover, peak lower limb force production on the wall contributes to optimize flip turn time [35]. On the other hand, muscle endurance is required to minimize the loss of stroke efficiency [6]. It is possible that the level of hypohydration created in this study was too low to impact in any meaningful way lower limb muscle strength and endurance and, therefore, swimming performance. Anaerobic contribution has been demonstrated to be important for swimming performance [5]. However, it has been demonstrated that the glycolytic pathway is not impacted in a significant manner by decreased cell volume and water content [36].

This study has limitations. First, the quality and validity of our observations are tributary to the intrinsic motivation and desire of swimmers to produce a maximal effort during the 100 m front crawl, including the start on the block and the push from the wall during the flip turn. Second, the results only apply to university-level competitive swimmers. Third, per the research design, participants were not blinded to the hydration conditions. Therefore, that a nocebo effect contributed to decrease performance in some swimmers cannot be ruled out [37]. Fourth, with nine participants, appropriate counterbalancing of the conditions was not achieved. However, no order effect was observed for any performance-related outcomes. Fifth, no measurements of blood/plasma sodium or osmolality were performed. Therefore, it cannot be ruled out that, although swimmers had lost 1.5% of their body mass and were considered hypohydrated based on urinary markers, hydration status was within the physiological range based on blood/plasma-related variables [14]. Finally, swimming performance times were not measured using an electronic timing system. As a result, small performance changes may have been missed, although it must be noted that perfect inter-rater reliability was achieved and that we used a timing protocol supported by FINA.

## 6. Conclusions

The present results indicate that mild hypohydration of 1.5% body mass does not impact 100 m front crawl performance time nor force production on the starting block, thereby suggesting that resorting to a strategy aimed at optimizing hydration prior to such an exercise is not necessary and that ad libitum drinking is likely just what is needed.

## Figures and Tables

**Table 1:** Physical, physiological, anthropometric and training characteristics of participants.

Characteristics	Mean ± SD
Age (year)	22 ± 2
Height (cm)	174 ± 6
Body mass (kg)	71 ± 9
Fat mass (%)	18 ± 8
Body mass index (kg/m^2^)	23.5 ± 2.4
Lean mass (%)	82 ± 8
Lower limb lean mass (kg)	21 ± 3
Resting systolic blood pressure (mmHg)	123 ± 9
Resting diastolic blood pressure (mmHg)	71 ± 8
Resting heart rate (beats/min)	68 ± 11
Training experience (year)	10 ± 3
Training volume (h/week)	17 ± 7
Personal best on 100 m front crawl (long-course) (s)	60.4 ± 3.4
Ratio of personal best time/world record time	1.29 ± 0.07

SD: standard deviation.

**Table 2:** Perceived thirst and perceived heat stress immediately before heat exposure, after heat exposure, post-heat exposure and prior to testing while being euhydrated and hypohydrated. Values are means ± SD.

Variables (AU)	Hydration Conditions	Time-Period			
		Pre-HE	Post-HE	1 h Post-HE	Pre-testing
Perceived thirst	Euhydrated	3.3 ± 0.9	4.7 ± 2.3	2.9 ± 1.6	3.9 ± 1.5 ^*¶&^
	Hypohydrated	3.3 ± 1.0	6.4 ± 1.5	6.1 ± 1.2	5.1 ± 1.6
Perceived heat stress	Euhydrated	3.9 ± 1.3	5.1 ± 1.4	2.8 ± 0.9	3.1 ± 1.1 *
	Hypohydrated	3.6 ± 0.8	5.5 ± 1.2	3.3 ± 0.7	3.3 ± 1.0

*: time effect; ¶: condition effect; &: interaction effect. AU: arbitrary units; Pre-HE: pre-heat exposure; Post-HE: post-heat exposure.
